# Sequential Evaluation of Hematology Markers as a Prognostic Factor in Glioblastoma Patients

**DOI:** 10.3390/biomedicines12051067

**Published:** 2024-05-12

**Authors:** João Meira Gonçalves, Bruno Carvalho, Rui Tuna, Patricia Polónia, Paulo Linhares

**Affiliations:** 1 Neurosurgery Department, Centro Hospitalar Universitário São João, 4200-319 Oporto, Portugal; 2 Faculty of Medicine, Oporto University, 4200-319 Oporto, Portugal; 3 Neurosciences Centre, Hospital CUF, 4099-001 Oporto, Portugal

**Keywords:** glioblastoma, monocyte-to-lymphocyte ratio, neutrophils-to-lymphocyte ratio, overall survival, platelet-to-lymphocyte ratio, prognostic markers, hematology markers

## Abstract

In our study, we investigated the prognostic significance of hematological markers—NLR (Neutrophil-to-Lymphocyte Ratio), PLR (Platelet-to-Lymphocyte Ratio), and RDW-CV (Red Blood Cell Distribution Width—Coefficient of Variation)—in 117 glioblastoma patients. The data collected from January 2016 to December 2018 included demographics, clinical scores, and treatment regimens. Unlike previous research, which often examined these markers solely before surgery, our unique approach analyzed them at multiple stages: preoperative, postoperative, and before adjuvant therapies. We correlated these markers with the overall survival (OS) and progression-free survival (PFS) using statistical tools, including ANOVA, Cox regression, and Kaplan–Meier survival analyses, employing SPSS version 29.0. Our findings revealed notable variations in the NLR, PLR, and RDW-CV across different treatment stages. The NLR and PLR decreased after surgery, with some stabilization post-STUPP phase (NLR: *p* = 0.007, η^2^p = 0.06; PLR: *p* = 0.001, η^2^p = 0.23), while the RDW-CV increased post-surgery and during subsequent treatments (RDW-CV: *p* < 0.001, η^2^p = 0.67). Importantly, we observed significant differences between the preoperative phase and other treatment phases. Additionally, a higher NLR and RDW-CV at the second-line treatment and disease progression were associated with an increased risk of death (NLR at 2nd line: HR = 1.03, *p* = 0.029; RDW-CV at progression: HR = 1.14, *p* = 0.004). We proposed specific marker cut-offs that demonstrated significant associations with survival outcomes when applied to Kaplan–Meier survival curves (NLR at 2nd line < 5: *p* < 0.017; RDW-CV at progression < 15: *p* = 0.007). An elevated NLR and RDW-CV at later treatment stages correlated with poorer OS and PFS. No significant preoperative differences were detected. These biomarkers may serve as non-invasive tools for glioblastoma management.

## 1. Introduction

The diagnostic approach to gliomas has experienced a significant transformation due to recent revisions in the new World Health Organization (WHO) classification. The term “glioblastoma” (GBM) is now specifically assigned to IDH-wildtype tumors, and its diagnosis hinges on histological features [1]. GBM is the most aggressive form of adult-type diffuse gliomas. This classification is typically given when the tumor does not display mutations in the IDH (isocitrate dehydrogenase) genes. On the other hand, IDH-mutant type refers to tumors characterized by mutations in the IDH genes, which include IDH1 and IDH2 and generally present a better prognosis [1,2,3].

The determination of prognosis in gliomas has been a critical aspect influencing treatment approaches. A decision-analytic method to define poor prognosis, as discussed by van Dijk et al. (2008), highlights the importance of adapting treatments based on prognostic markers. This methodology aids in identifying patients who may profit from more aggressive interventions while sparing those with a better prognosis from burdensome treatments [2].

Newly diagnosed cases of glioblastoma present a challenging medical task, characterized by a five-year survival rate of only 7.2% [3]. Some of the identified factors associated with poor survival outcomes in glioblastoma patients include increasing age, poor performance status (PS), and corticosteroid use [4].

Present studies highlight the importance of distinct blood markers, including the Neutrophil-to-Lymphocyte Ratio (NLR), the Platelet-to-Lymphocyte Ratio (PLR), and the Red Cell Distribution Width (RDW), as predictive factors for the progression of diverse tumors. The NLR and PLR aid as indicators of the body’s systemic inflammatory response, and ongoing research has investigated their associations with different cancer types, particularly focusing on colorectal and oropharyngeal cancers [5]. Additionally, the RDW which quantifies red blood cell size variability, is linked to cancer prognosis. Studies have found that elevated RDW levels were more common in cancer patients who did not survive, highlighting its significance as an indicator of prognosis [6].

Subsequently, different studies are highlighting the prognostic applicability of the NLR and PLR in the context of glioblastoma. The findings from these studies have consistently revealed that elevated levels of the NLR and PLR before treatment are associated with less favorable outcomes in these patients. Notably, the NLR seems to be a more decisive prognostic marker than the PLR in certain studies [7,8,9].

In the present study, we gathered data on the NLR, PLR, and Red Cell Distribution Width-Coefficient of Variation (RDW-CV) at various stages including pre-operatively, post-operatively, and prior to specific adjuvant treatments. Our objective was to investigate the potential influence of these hematological markers on Overall Survival (OS) and Progression-Free Survival (PFS) in glioblastoma patients, thereby contributing to a deeper understanding of their prognostic significance in this context.

## 2. Materials and Methods

This study aims to evaluate whether the values of the NLR, PLR, and RDW-CV at different treatment time points can predict outcomes in patients with glioblastomas. 

It is a retrospective study, utilizing data collected from clinical records between 1 January 2016 and 31 December 2018 at Centro Hospitalar de São João in Porto.

The data gathered include patient demographics (age and sex), preoperative Eastern Cooperative Oncology Group (ECOG) and Karnofsky Performance Scale (KPS) scores, date of glioblastoma diagnosis (marked by the first CT scan), presence of significant preoperative deficits, and whether the lesion was in an eloquent area (responsible for critical functions, such as language, sensory processing, motor skills, vision, and cognition). We also conducted a review of medical records to determine whether the patients were undergoing corticosteroid therapy prior to their surgical procedures, leading to the identification of this patient cohort.

Additionally, NLR, PLR, and RDW-CV values were recorded at four specific time points: pre-surgery, pre-first progression after surgery, pre-first adjuvant therapy, and pre-second adjuvant therapy. Most patients in our department were subjected to the Stupp protocol, which consists of a standard first-line treatment approach. This protocol typically involves radiotherapy and concurrent temozolomide chemotherapy. For second-line or adjuvant treatment options, a combination involving bevacizumab was mainly used. The NLR was calculated by dividing the absolute count of neutrophils by the absolute count of lymphocytes, serving as a marker of inflammation and immune response. The PLR, on the other hand, was determined by dividing the absolute count of platelets by the absolute count of lymphocytes. Previous articles focus on the prognostic role of the RDW generally without specifying which measure (RDW-CV or Red Cell Distribution Width—Standard Deviation (RDW-SD)) was used. Our choice fell on the RDW-CV, a parameter that establishes a ratio of the Red Cell Distribution Width to the mean corpuscular volume, thereby providing a comparative measure of the variation in red blood cell sizes.

Information regarding the type of surgery (biopsy, total surgical removal, or partial removal), first and second-line adjuvant treatments, and the use of corticosteroids prior to surgery was also collected. The key outcomes measured were the dates of reoperation, tumor progression, death, OS, and PFS. Tumor progression was defined using criteria such as Response Assessment in Neuro-Oncology (RANO), which was applied to patients who underwent surgery and initially experienced tumor progress. Overall Survival was defined as the duration from either the diagnosis or initiation of treatment to the time of death from any cause. Progression-free survival is measured from the start of treatment until the observed disease progression or death from any cause, whichever occurs first. 

The study included patients diagnosed with glioblastoma IDH-wildtype as per the WHO 2016 classification, aged 18 years or older, who underwent surgery and adjuvant treatment. It excluded patients with IDH mutant glioblastomas, missing data, surgeries predating 2016, or a history of other major diseases that could affect inflammatory markers, like previous cancer treatments, infections, or acute inflammation. This study’s limitations include the retrospective nature, which might impact data completeness and quality, and the potential for selection and recall biases. The generalizability of the findings might also be limited due to these factors.

The expected outcomes include identifying whether high or low levels of NLR, PLR, and RDW are significant in predicting OS, their correlation with the cancer stage, and evaluating their combinatorial prognostic value.

The data were analyzed using SPSS, version 29.0 (IBM Corp., Armonk, NY, USA, 2023). The descriptive statistics are presented as the means and standard deviations for the normally distributed variables and the medians and quartiles otherwise. For the categorical variables, frequencies and percentages are presented. Repeated measures ANOVA with Sidak multiple comparisons tests were used to analyze the variation of the NLR, PLR, and RDW-CV along the four treatment phases. In addition to the RM-ANOVA, univariate Cox regression analyses were performed to assess the impact of these hematological markers on the OS and PFS. Survival analysis included Kaplan–Meier survival curves and log-rank tests and was used to further delineate the prognostic significance of these biomarkers, with the proposed cut-offs for the NLR and PLR validated against the survival outcomes. Adjusted Cox regressions were then conducted, controlling for relevant covariates such as age, ECOG performance status, preoperative neurological deficit, type of surgery, and corticosteroid use. Log-minus-log plots were checked to verify Cox proportional hazard assumptions. Significance was deemed for *p* < 0.05.

The nature of our research, being retrospective and involving the analysis of pre-existing data under confidentiality protocols, has been carefully evaluated by the Ethical Committee of our hospital. It was determined that formal ethical approval was not a requisite for this study, based on its retrospective design and the fact that it solely involved the analysis of anonymized data, thereby perpetuating the principles of confidentiality.

## 3. Results

In this study, we analyzed data from 117 patients. The average age was 61.51 years (SD = 11.47), with an age range from 19 to 85 years. The patients’ characteristics are described in detail in Table 1. The prevalence of preoperative neurologic deficit was 65.0% (n = 76), and 78 (66.7%) patients were treated with corticosteroids in the preoperative period. The prevalence of tumor location in the eloquent area was 73.5% (n = 86). The mean ECOG performance status pre-operation was 1.03 (SD = 0.88), and the mean KPS pre-operation was 83.85 (SD = 15.36). The mean OS duration for the cohort was approximately 16.69 months, and the average PFS duration was 8.25 months. Re-operations were carried out in seven patients (6%), with an average time to reoperation of approximately 7.03 months from the initial diagnosis.

In our cohort, patients undergoing Gross Total Removal without preoperative neurological deficits or corticosteroid therapy demonstrated an average OS of 19.68 months and an average PFS of 10.70 months. 

**Figure 1:**
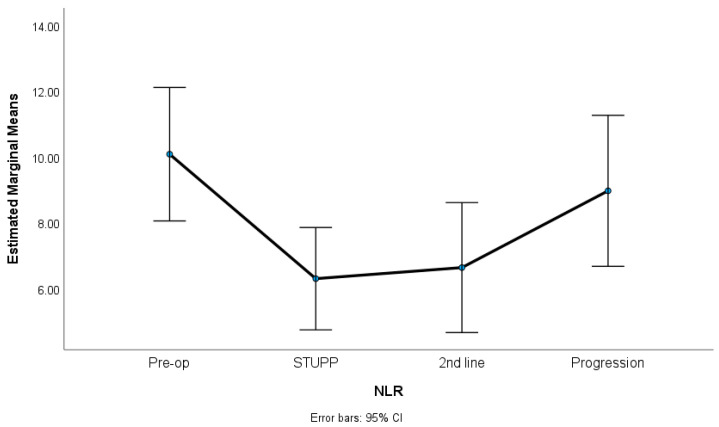
NLR variation along treatment phases.

**Figure 2:**
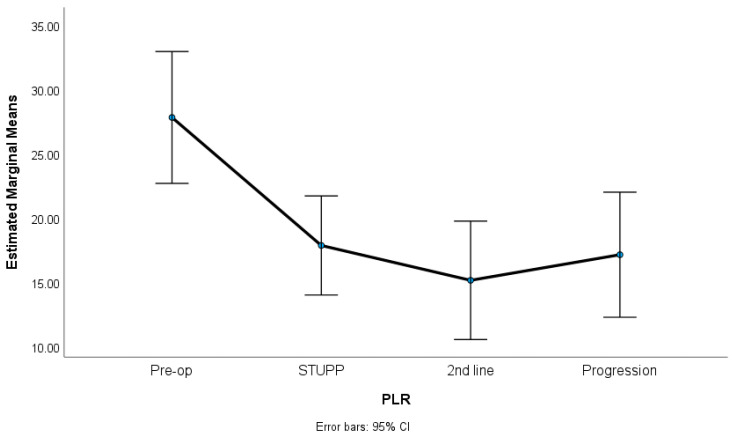
PLR variation along treatment phases.

**Figure 3:**
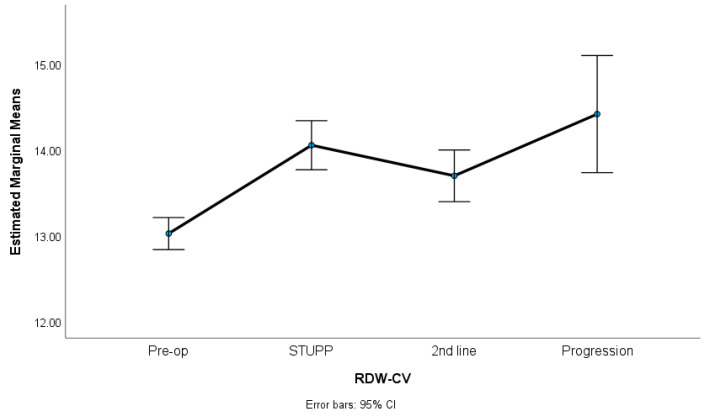
RDW-CV variation along treatment phases.

In this study, we observed significant variations in key hematological parameters. Specifically, the NLR and PLR showed noteworthy changes across different treatment phases, as revealed by our statistical analysis. Figure 1, Figure 2 and Figure 3 show NLR, PLR, and RDW-C variation along the treatment phases. The NLR and PLR decreased after surgery, with some stabilization after the STUPP phase. The RDW-CV increased after surgery, showing a positive slope, despite a slight decrease during second-line treatment. Table 2 shows the means, standard errors, and repeated measures ANOVA tests. A total of 62 patients were included in this analysis. The missing were listwise excluded.

The results indicated significant differences among the treatment phases for the NLR (*p* = 0.007, η^2^p = 0.06), the PLR (*p* = 0.001, η^2^p = 0.23), and the RDW-CV (*p* < 0.001, η^2^p = 0.67). The effect sizes (partial eta-squared) ranged from medium to high, suggesting a substantive impact of the treatment phases on the variability of these parameters. Sidak multiple comparisons tests showed that the preoperative phase was significantly different from the other treatment phases. These phases were not statistically different among themselves.

**Figure 4:**
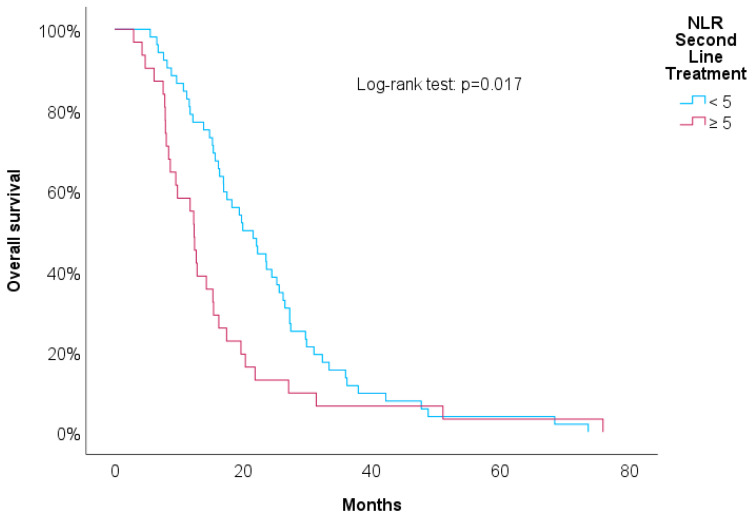
Kaplan–Meier survival curves for OS compared to NLR at second line of treatment.

**Figure 5:**
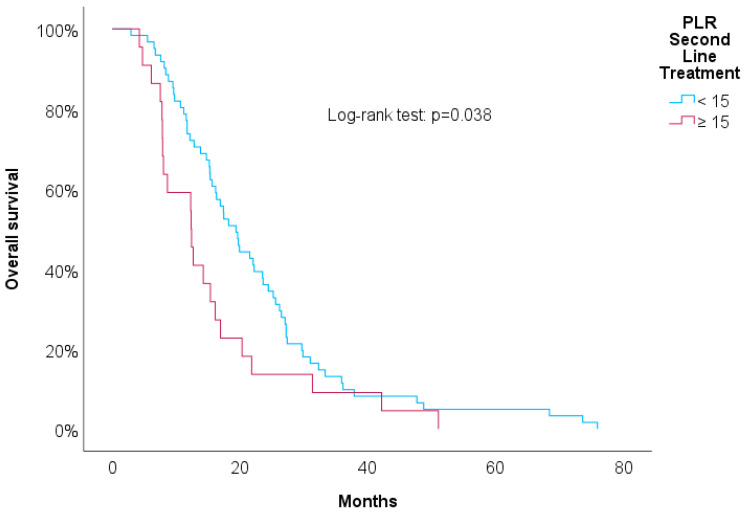
Kaplan–Meier survival curves for OS compared to PLR at second line of treatment.

**Figure 6:**
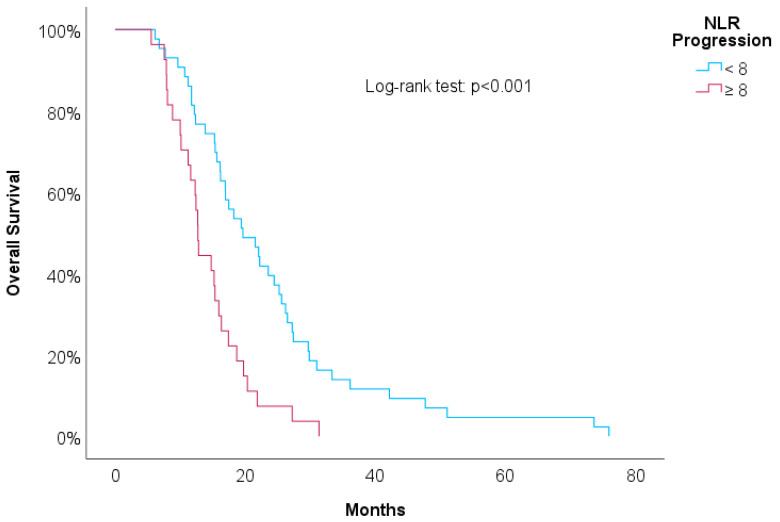
Kaplan–Meier survival curves for OS compared to NLR at progression.

**Figure 7:**
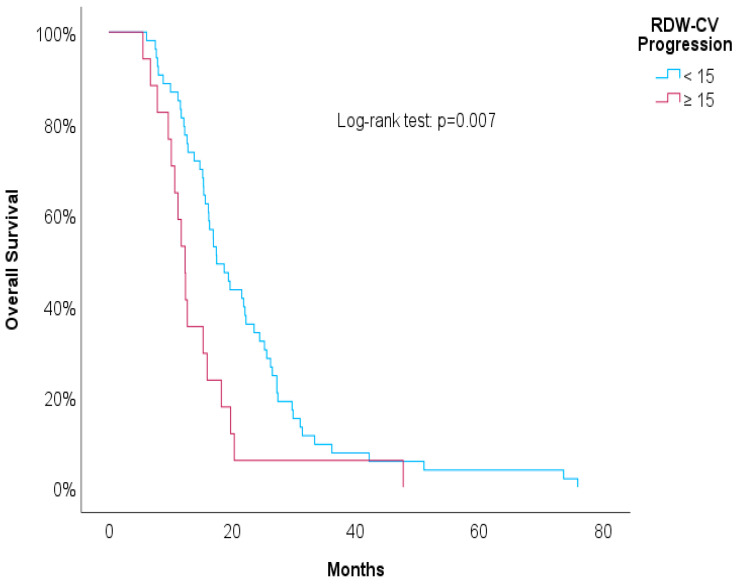
Kaplan–Meier survival curves for OS compared to RDW-CV at progression.

Table 3 shows univariate Cox regressions for the OS and PFS. Cut-offs were proposed for significant associations. An increased risk of death was associated with a higher NLR at the second line of treatment (HR = 1.03, *p* = 0.029), NLR at progression (HR = 1.04, *p* = 0.006), and RDW-CV at progression (HR = 1.14, *p* = 0.004). A result close to statistical significance was detected in the PLR at the second line of treatment (HR = 1.01, *p* = 0.084). For these variables, we proposed cut-offs that were statistically significant when implemented in Kaplan–Meier survival curves. Figure 4, Figure 5, Figure 6 and Figure 7 show increased overall survival for the NLR at the second line of treatment < 5 (*p* < 0.017), for the PLR at the second line of treatment <15 (*p* = 0.038), for the NLR at progression (*p* < 0.01), and for the RDW-CV at progression (*p* = 0.007).

Table 4 presents the results of adjusted Cox regressions for the proposed cut-offs adjusted for the following covariates: age, ECOG, preoperative neurologic deficit, surgery, and preoperative corticosteroid use. After adjusting for the covariates, all the predictors maintained the association with overall survival. An NLR ≥ 5 at the second line of treatment was associated with increased risk of mortality at t + 1 at the rate of 88% more risk (aHR = 1.88, *p* = 0.008), a PLR ≥ 5 at the second line of treatment was associated with almost twice the risk of mortality at t + 1 (aHR = 1.93, *p* = 0.012), an NLR ≥ 15 at progression was associated with 2.22 times more risk of mortality at t + 1 (aHR = 2.22, *p* = 0.004), and an RDW-CV ≥ 8 at progression was associated with 2.30 times more risk of mortality at t + 1 (aHR = 2.30, *p* = 0.013). Patients with a higher KPS were associated with a lower risk of mortality (HR = 0.98, *p* = 0.001, 95% CI = [0.96–0.99]).

## 4. Discussion

Our research on the Sequential Evaluation of Hematology Markers as a Prognostic Factor in Glioblastoma Patients has focused exclusively on a specific cohort of patients. We intentionally excluded glioblastomas with IDH mutations, directing our attention to the cohort of patients with IDH-wildtype glioblastomas, which are known to have a poorer prognosis and represent a similar tumor grade. We also acknowledge the diverse glioblastoma populations that have undergone various types of surgical interventions, specifically biopsy or partial resection. These procedures are recognized as prognostic influencers, impacting patient outcomes differently. Lastly, our study has taken into consideration the administration of corticosteroids before the calculation of NLR, PLR, and RDW values, recognizing these as potential confounding variables. Most notably, our study differentiates itself by examining the levels of these markers not just before surgery but also during the complete treatment course of glioblastomas. This includes periods during which, patients were undergoing first-line or second-line adjuvant treatments. By tracking these markers throughout the disease, we aim to provide a more comprehensive understanding of their prognostic value and their potential fluctuations in response to different therapeutic interventions.

The study in question involved the analysis of clinical data from 117 glioblastoma patients. There was a significant variation in the NLR, PLR, and RDW-CV throughout different treatment phases. After surgery, a decrease in the NLR and PLR was observed, while the RDW-CV levels increased. The analysis further revealed that higher levels of the NLR and RDW-CV at the point of disease progression and at the second-line adjuvant treatment phase were associated with an increased risk of mortality. Specifically, during the second line of treatment phase, patients with an NLR of 5 or higher and a PLR of 15 or higher were associated with a worse outcome. On the other hand, as glioblastoma progressed after surgery, an NLR of 8 or higher and an RDW-CV of 15 or higher were linked to a poorer prognosis. After adjusting for covariates, all the predictors maintained the association with overall survival.

Recent studies corroborate the prognostic significance of elevated levels of the NLR and PLR before surgery [10,11,12]. Conversely, the role of the RDW as a prognostic indicator has been a subject of divergence. While previous studies emphasized an association between increased RDW levels and a poorer prognosis in GBM patients, recent investigations have presented a different perspective. A comprehensive analysis, based on a substantial sample size, has suggested a lack of direct correlation between the RDW and OS in GBM patients, even though there is a temporal evolution of the RDW that exhibits an upward trajectory over the course of the disease [13]. Finally, there is increasing evidence, based on studies using large sample sizes, that suggests that both a high pre-treatment and preoperative NLR are correlated with poor overall survival in GBM patients. This aligns with the conclusions from our results [11,12].

Ongoing investigations have concentrated on the role of inflammatory indicators such as counts of neutrophils, lymphocytes, and platelets as well as ratios like the NLR and the PLR, which are readily accessible through routine bloodwork. Nonetheless, the significance of these markers in predicting glioblastoma outcomes remains a subject of controversy. The contribution of persistent inflammation to the onset and expansion of cancer is recognized, particularly as it facilitates tumor development and affects vascularization. Typically, individuals with glioblastomas develop an increase in neutrophils and a reduction in lymphocytes, a condition often generated from the excessive production of G-CSF by the tumors, which in turn tips blood cell production in favor of granulocytes [14]. An increased level of neutrophils and an elevated NLR have been associated with unfavorable prognoses in several types of cancer, GBM included. Likewise, the cell count of lymphocytes has not been a consistent prognostic tool for GBM, possibly because of the variation in lymphocyte types [9]. High platelet levels can promote tumor growth and angiogenesis, which is the formation of new blood vessels. Platelets can release growth factors like Vascular Endothelial Growth Factor (VEGF), which is crucial for angiogenesis, thereby potentially aiding tumor growth and spread [9]. The PLR may be suggestive of a prothrombotic state often associated with tumor growth and metastasis. Platelets can shield tumor cells from immune detection and destruction, promoting dissemination [11,12,15,16].

Patients with glioma and higher RDW levels tend to have a less favorable prognosis. The underlying reasons for this correlation could be multifaceted, involving complex interactions between the RDW and various inflammatory markers, cytokines like IL-6 and tumor necrosis factor-alpha, which are known to influence the behavior of tumor cells [17,18]. Additionally, increased RDW levels can lead to enhanced production of reactive oxygen species and contribute to tissue hypoxia, which may further complicate postoperative recovery and increase the likelihood of complications [19]. The rise in RDW can be a consequence of elevated inflammatory cytokine levels, which in turn may lead to increased hepcidin production, affecting red blood cell synthesis and potentially leading to anemia. In patients with glioma, a higher RDW could reflect several underlying issues, such as inflammation, oxidative stress, and poor nutritional status, all of which can adversely affect the patient’s health and treatment outcomes [7,20].

The incorporation of such biological markers opens the door to more personalized medical approaches. A new prognostic model was designed using a random forest approach, based on preoperative peripheral blood markers, aimed at predicting three-year survival for GBM patients undergoing surgery and the STUPP regimen. The model includes several variables, notably indices of inflammation and nutritional status [12].

The dynamic changes in these markers throughout different treatment phases can inform clinicians about the patient’s response to therapy. For instance, a post-surgical decrease in the NLR and PLR may be indicative of a positive response to surgery. In contrast, increases in the RDW-CV might necessitate closer monitoring. Secondly, the association of higher levels of the NLR and RDW-CV with increased mortality risk can guide the intensity and type of adjuvant therapy. Patients with elevated levels of these markers might benefit from more aggressive or alternative treatment modalities. Thirdly, during the second line of treatment, the presence of high NLR and PLR levels could be used to identify patients at a higher risk of a worse outcome, potentially prompting earlier intervention or palliative care. Lastly, these markers could facilitate more personalized follow-up strategies, where patients with a higher NLR and RDW-CV post-surgery might require more frequent imaging and clinical evaluation to detect early signs of progression.

Even though serum biomarkers such as eosinophils, the Systemic Immune-Inflammation Index (SII), and the Systemic Inflammation Response Index (SIRI) also play an important role in the interaction between tumorigenesis and the immune system, they were not analyzed in this study. Higher eosinophil counts have been associated with better prognoses in GBM. Conversely, the SII and the SIRI, which indicate a predominant inflammatory response, are often associated with poorer outcomes [21]. Cell-free DNA (cfDNA) consists of DNA fragments released from cells undergoing cell death. A specific subset of cfDNA, known as circulating tumor DNA (ctDNA), is currently under study. Recent meta-analyses have highlighted a significant finding: higher levels of cfDNA are correlated with a greater risk of adverse outcomes. The analyses included comparative studies where cerebrospinal fluid ctDNA demonstrated superior biomarker performance (Area Under Curve, AUC = 0.947) compared to plasma ctDNA (AUC = 0.741), suggesting its potential for more accurate prognostic assessments [22].

Retrospective analyses are inherently subject to selection bias. The patients included may not represent the entire spectrum of glioblastoma cases, as those with missing data, surgeries predating 2016, and patients with IDH mutation and other major diseases affecting inflammatory markers were also excluded. This selection could twist the results, as these factors could significantly alter the levels of the markers being studied. The single-center nature of the study may limit the applicability of the results to other settings. Variations in patient demographics, treatment protocols, and healthcare delivery systems could influence the biomarkers differently across institutions or populations such as the type of first-line or second-line adjuvant type treatment commonly applied in our department.

Lastly, this research focused on two essential partially unanswered questions in the past years. First, it explored the intricate relationship between the hematological markers the NLR, the PLR, and the RDW-CV and the patient’s response to treatment. This aspect of the analysis aimed to recognize patterns that could potentially guide the personalization of therapeutic approaches, allowing for treatments to be more finely adjusted to the individual’s biological answer as shown by these markers. The second area of focus was the longitudinal variability of these indicators throughout the disease course, including throughout treatment. By assessing how the NLR, PLR, and RDW fluctuated over time, we sought to gain perceptions of the disease’s progression and the effectiveness of treatment modalities [23]. This longitudinal analysis is essential, as it may uncover temporal patterns that might serve as early indicators of treatment success or failure and could potentially predict disease recurrence or progression.

## 5. Conclusions

Our study presented a detailed analysis of the prognostic significance of hematological markers such as the NLR, PLR, and RDW-CV in patients with IDH-wildtype glioblastoma. We observed that variations in these markers at different treatment phases—specifically elevated levels of NLR and RDW-CV at the onset of second-line treatment and progression—were significantly associated with a decreased OS and PFS. We did not find statistically significant differences in the preoperative marker levels. These findings highlight the potential of the NLR, PLR, and RDW as accessible, non-invasive prognostic tools in the clinical management of glioblastoma. Future studies should focus on validating these markers in larger, multi-center cohorts and exploring the essential biological mechanisms driving their involvement with patient outcomes. Finally, understanding these dynamics could lead the way for more personalized and timely interventions, improving survival and quality of life for glioblastoma patients.

## Figures and Tables

**Table 1:** This table presents a detailed summary of various patient characteristics and their association with median overall survival (mOS). The variables assessed include gender, tumor location, tumor size, the extent of resection (Gross Total Resection, GTR), standard treatment adherence (Stupp protocol), and IDH-1^R132H^ mutation status. The number of patients under each category is provided, along with median overall survival times expressed in months and the corresponding 95% Confidence Intervals (CIs). Hazard Ratios (HRs) with 95% CIs are also provided, along with *p*-values.

Variables	No.	mOS (95% CI) Months	HR (95% CI)	*p*
Gender				
Female	40	12.5 (7.5–17.5)	1.18 (0.82–1.68)	0.380
Male	77	13.0 (10.8–15.2)		
Location				
Frontal	19	12.2 (9.0–15.4)	1.05 (0.94–1.17)	0.500
Temporal	15	17.2 (10.5–23.9)		
Parietal	7	11.2 (5.0–17.4)		
Other location	11	8.5 (3.1–13.9)		
Mixed	65	13.5 (9.6–17.3)		
Size				
≤4.5 cm	39	13.5 (8.0–19.0)	0.97 (0.68–1.36)	0.750
>4.5 cm	78	12.5 (10.5–14.5)		
Resection				
GTR	51	13.5 (10.7–16.3)	1.49 (1.04–2.11)	0.029
Non-GTR	66	9.5 (6.0–13.0)		
Standard Treatment				
Yes	67	15.0 (12.2–17.8)	2.45 (1.68–3.55)	0.000
No	50	8.0 (4.5–11.5)		
IDH-1R132H				
Mutant	18	17.5 (9.0–26.0)	1.62 (1.02–2.56)	0.040
Wildtype	99	12.2 (9.5–14.9)		

**Table 2:** RM-ANOVA for NLR, PLR, and RDW-CV variation along treatment phases. Results presented as means (standard errors); η^2^_p_, partial eta-squared with thresholds 0.01 (low), 0.06 (medium) and 0.14 (high); (a) preoperative vs. STUPP (*p* = 0.032)/2nd line treatment (*p* = 0.048); (b) preoperative vs. STUPP (*p* = 0.007)/2nd line treatment (*p* = 0.002)/progression (*p* = 0.014); (c) preoperative vs. STUPP (*p* < 0.001)/2nd line treatment (*p* < 0.001)/progression (*p* < 0.001).

n = 62	Preoperative	STUPP	2nd Line Treatment	Progression	RM-ANOVA	Sidak Tests
NLR	10.08 (1.01)	6.30 (0.78)	6.63 (0.99)	8.96 (1.15)	*p* = 0.007 (η^2^_p_ = 0.06)	(a)
PLR	27.81 (2.56)	17.87 (1.92)	15.17 (2.30)	17.15 (2.43)	*p* = 0.001 (η^2^_p_ = 0.23)	(b)
RDW-CV	13.02 (0.09)	14.05 (0.14)	13.70 (0.15)	14.42 (0.34)	*p* < 0.001 (η^2^_p_ = 0.67)	(c)

**Table 3:** Univariate Cox regressions for overall survival and progression-free survival. Results presented as Hazard Ratios (HR), 95% Confidence Intervals (95% CI), and *p*-values.

	HR	95% CI	*p*-Value	Cut-Off Proposal
Overall Survival				
Preoperative				
NLR (n = 117)	0.99	0.96–1.02	0.509	-
PLR (n = 117)	0.99	0.98–1.00	0.218	-
RDW-CV (n = 117)	1.02	0.81–1.28	0.864	-
STUPP				
NLR (n = 101)	1.00	0.96–1.03	0.779	-
PLR (n = 101)	0.99	0.98–1.01	0.335	-
RDW-CV (n = 102)	0.96	0.81–1.13	0.606	-
2nd line treatment				
NLR (n = 83)	1.03	1.00–1.06	0.029	≥5
PLR (n = 83)	1.01	1.00–1.02	0.084	≥15
RDW-CV (n = 83)	1.04	0.88–1.24	0.629	-
Progression				
NLR (n = 70)	1.04	1.01–1.06	0.006	≥8
PLR (n = 70)	1.01	1.00–1.02	0.162	-
RDW-CV (n = 70)	1.14	1.05–1.24	0.003	≥15
Progression-free survival				
NLR (n = 117)	1.00	0.97–1.03	0.824	-
PLR (n = 117)	1.00	0.99–1.01	0.716	-
RDW-CV (n = 117)	1.03	0.81–1.31	0.800	-

**Table 4:** Adjusted Cox regressions for proposed cut-offs adjusted for covariates. Results presented as adjusted Hazard Ratios (aHR), 95% Confidence Intervals (95% CI), and *p*-values; all Cox regressions adjusted for age, ECOG, preoperative neurologic deficit, surgery, and preoperative corticosteroid use.

	aHR	95% CI	*p*-Value
Overall Survival			
2nd line treatment			
NLR ≥ 5 (n = 83)	1.88	1.17–3.01	0.009
PLR ≥ 15 (n = 83)	1.93	1.16–3.21	0.012
Progression			
NLR ≥ 15 (n = 70)	2.22	1.28–3.83	0.004
RDW-CV ≥ 8 (n = 70)	2.30	1.19–4.41	0.013

## Data Availability

The data that support the findings of this study are available from the corresponding author upon reasonable request.
